# Modified Predictive Model and Nomogram by Incorporating Prebiopsy Biparametric Magnetic Resonance Imaging With Clinical Indicators for Prostate Biopsy Decision Making

**DOI:** 10.3389/fonc.2021.740868

**Published:** 2021-09-13

**Authors:** Jin-feng Pan, Rui Su, Jian-zhou Cao, Zhen-ya Zhao, Da-wei Ren, Sha-zhou Ye, Rui-da Huang, Zhu-lei Tao, Cheng-ling Yu, Jun-hui Jiang, Qi Ma

**Affiliations:** ^1^Medical School, Ningbo University, Ningbo, China; ^2^Comprehensive Urogenital Cancer Center, Ningbo First Hospital, The Affiliated Hospital of Ningbo University, Ningbo, China; ^3^Department of Urology, Ningbo First Hospital, The Affiliated Hospital of Ningbo University, Ningbo, China; ^4^Ningbo Clinical Research Center for Urological Disease, Ningbo, China; ^5^Department of Radiology, Ningbo First Hospital, The Affiliated Hospital of Ningbo University, Ningbo, China; ^6^Translational Research Laboratory for Urology, the Key Laboratory of Ningbo City, Ningbo First Hospital, The Affiliated Hospital of Ningbo University, Ningbo, China

**Keywords:** prostate cancer, prostate biopsy, prostate-specific antigen, biparametric MRI (Bp-MRI), PIRADS score, prostate-specific antigen density (PSAD)

## Abstract

**Purpose:**

The purpose of this study is to explore the value of combining bpMRI and clinical indicators in the diagnosis of clinically significant prostate cancer (csPCa), and developing a prediction model and Nomogram to guide clinical decision-making.

**Methods:**

We retrospectively analyzed 530 patients who underwent prostate biopsy due to elevated serum prostate specific antigen (PSA) levels and/or suspicious digital rectal examination (DRE). Enrolled patients were randomly assigned to the training group (*n* = 371, 70%) and validation group (*n* = 159, 30%). All patients underwent prostate bpMRI examination, and T2-weighted imaging (T2WI) and diffusion-weighted imaging (DWI) sequences were collected before biopsy and were scored, which were respectively named T2WI score and DWI score according to Prostate Imaging Reporting and Data System version 2 (PI-RADS v.2) scoring protocol, and then PI-RADS scoring was performed. We defined a new bpMRI-based parameter named Total score (Total score = T2WI score + DWI score). PI-RADS score and Total score were separately included in the multivariate analysis of the training group to determine independent predictors for csPCa and establish prediction models. Then, prediction models and clinical indicators were compared by analyzing the area under the curve (AUC) and decision curves. A Nomogram for predicting csPCa was established using data from the training group.

**Results:**

In the training group, 160 (43.1%) patients had prostate cancer (PCa), including 128 (34.5%) with csPCa. Multivariate regression analysis showed that the PI-RADS score, Total score, f/tPSA, and PSA density (PSAD) were independent predictors of csPCa. The prediction model that was defined by Total score, f/tPSA, and PSAD had the highest discriminatory power of csPCa (AUC = 0.931), and the diagnostic sensitivity and specificity were 85.1% and 87.5%, respectively. Decision curve analysis (DCA) showed that the prediction model achieved an optimal overall net benefit in both the training group and the validation group. In addition, the Nomogram predicted csPCa revealed good estimation when compared with clinical indicators.

**Conclusion:**

The prediction model and Nomogram based on bpMRI and clinical indicators exhibit a satisfactory predictive value and improved risk stratification for csPCa, which could be used for clinical biopsy decision-making.

## Introduction

PCa is the second leading cause of cancer death in American men (1). An estimated 174,650 new cases were diagnosed in America in 2019, and 31,620 men are likely to die due to this malignant disease (2). Serum PSA is widely used in PCa screening due to its high diagnostic sensitivity and low testing cost (3). However, decades of clinical experience have shown that PSA is not ideal in terms of specificity, often leading to either overdiagnosis or overtreatment (4–6). Prostate biopsy is the most valuable method in the diagnosis of PCa (7, 8). Although preoperative preparation is actively improved, hemospermia, hematuria, and urinary tract infection are still the main adverse effects (9). Therefore, development of specific biomarkers or diagnostic tools for PCa is necessary (4, 10).

Multiparametric MRI (mpMRI) is the optimum imaging technology for the diagnosis and monitoring of PCa (11). Prebiopsy mpMRI scan has also shown promising results in PCa risk stratification (12). The Prostate Imaging Reporting and Data System (PI-RADS) is the most accepted guideline for the assessment of PCa based on mpMRI (13). However, widespread adoption of the mpMRI requires long acquisition time, incurs prohibitive costs, and increases the risk associated with contrast agents (14, 15). Thus, efforts have been made to introduce bpMRI, which uses shorter scanning time and without intravenous contrast agent. Several previous studies have compared the diagnostic accuracy of mpMRI and bpMRI, which suggested that the bpMRI is comparable to the mpMRI in the diagnosis of PCa and csPCa (16–18).

Though PSA and MRI are widely performed to diagnose PCa, both of them have certain false-negative rate and false-positive rate, especially if results are near the diagnostic threshold regions. Therefore, some studies attempt to combine MRI with clinical indicators to construct risk prediction models or Nomogram to improve the diagnostic accuracy of PCa (19). Niu et al. (20) designed a prediction model based on bpMRI that included age, PI-RADS score, and PSAD, and the sensitivity and specificity for the diagnosis of csPCa were 87.3% and 78.4%, respectively. Another single-center retrospective study constructed a diagnostic Nomogram also based on PSA, PSAD, and PI-RADS (bpMRI), with an AUC of 0.832 for csPSA (21).

Recent studies have shown that the combination of T2WI and DWI in bpMRI has satisfied accuracy in the detection of csPCa (16, 22). In this study, we established a novel bpMRI-based score system and constructed a more convenient prediction model in combination of this score system with clinical indicators. The purpose of the study is to evaluate whether our prediction model improves detection of csPCa and reduces the number of unnecessary biopsies. Furthermore, we constructed a Nomogram based on this model, and we believe that the Nomogram is effective and helpful for clinicians in biopsy decision-making.

## Patients and Methods

### Study Design and Participants

Between January 2018 and August 2020, a total of 618 men with clinical suspicion of PCa (PSA > 4 ng/ml and/or suspicious DRE results) underwent transrectal ultrasound (TRUS)-guided transperineal prostate biopsy in Ningbo First Hospital (this included both biopsy naive men as well as those with a prior negative biopsy). Inclusion criteria were PSA > 4 ng/ml and/or suspicious DRE result, and patients who underwent prostate bpMRI scan within 3 months prior to biopsy. Clinical indicators including age, tPSA, fPSA, f/tPSA, prostate volume (PV), and PSAD were also collected and measured. Exclusion criteria were treatment prior to biopsy (e.g., prostate surgery, chemotherapy, radiation therapy, or androgen deprivation therapy), bpMRI scan performed after biopsy, and incomplete data. Finally, a total of 530 patients were included in this study, 70% (*n* = 371) of the patients were randomly assigned to the training group, and 30% (*n* = 159) of the patients were assigned to the validation group. This study protocol was reviewed and approved by the Ethical Committee of Ningbo First Hospital (Ethical Approval Number: 2021B005). Written informed consent for prostate biopsy was routinely obtained before operation, and the need for informed consent of this study from patients was waived.

### bpMRI Protocol and Scoring Criteria

All bpMRI examinations were conducted with Siemens Magnetom Sonata 1.5-T superconducting MR scanner (sagittal, axial, and coronal T2WI: TR 3900 ms, TE 100 ms, FOV: 240×180, matrix: 240×160; DWI: TR 4900 ms, TE 89ms, FOV: 260×260, matrix: 100×124). The total scan time will be approximately 20 min. The routine scan protocol included T1WI, T2WI, and DWI (ADC) using body array coils (no endorectal coil). The maximum transverse diameter and anteroposterior diameter were measured in the transverse T2WI sequence, and the superoinferior diameter was measured in the sagittal T2WI sequence; PV was calculated by using the formula for prostate ellipsoid (transverse width × transverse length × longitudinal height × 0.52). Two experienced urological radiologists (Dr. ZYZ and Dr. DWR) who were blinded to pathological results and clinical information have analyzed and recorded the T2WI score and DWI score separately according to PI-RADS v.2 scoring protocol (23), and the comprehensive PI-RADS score was also performed on the bpMRI results. The different scores were discussed again until consensus was reached. Both the T2WI score and the DWI score of bpMRI results use a five-point scale based on findings on bpMRI performance. According to the PI-RADS v.2, we added the T2WI score and DWI score to obtain the Total score. The differences between PI-RADS v.2 score and Total score are summarized in **Supplementary Table S1**. In order to compare the diagnostic performance of PI-RADS score and Total score of bpMRI, they were separately included in multivariate analysis of the training group to determine independent predictors for PCa and csPCa.

### Prostate Biopsy and Histopathology and Definition

All biopsy operations were performed by the same experienced urological radiologist (Dr. RS) using a 7.5-MHz endocavity ultrasonic probe with color Doppler ultrasonography. Ten biopsy samples were obtained from each patient. If hypoechoic suspicious areas in peripheral zones or transition zones were found in MRI or ultrasound images, additional one to two targeted cores were also performed. All biopsy samples’ histopathological results were reported by Ningbo Pathology Center using the 2014 International Society of Urological Pathology (ISUP) modified Gleason grading system. A Gleason score of 3 + 4 or higher (ISUP ≥ 2) was defined as csPCa as the European Association of Urology Guidelines on Prostate Cancer suggested (24).

### Statistical Analysis

Patient characteristics were grouped according to biopsy results and summarized using descriptive statistics. Continuous variables are described as means ± standard deviations (SD) or medians and interquartile ranges (IQRs). Independent sample *t*-test was used for comparison between the csPCa group and the non-csPCa group. Multivariate regression analysis was used to determine the independent predictors for biopsy outcomes. Receiver operating characteristic (ROC) curve analysis and the AUC were used to examine the diagnostic performance of prediction models and clinical indicators. Spearman rank correlation was performed to analyze the relationship of categorical variables. AUC, sensitivity, specificity, negative predictive value (NPV), and positive predictive value (PPV) for each method were calculated and compared. The predictive accuracy of prediction models was validated using samples in the validation group to decrease the overfit bias. The best cutoff points for a biopsy threshold that balanced sensitivity and specificity was calculated using Youden’s J index (sensitivity + specificity − 1). ROC curves were compared by use of the method of DeLong et al. (25). Decision curve analysis (DCA) was performed and the greatest net benefit at a specific threshold probability was identified as that with the greatest clinical value. Further, Nomogram and calibration were also performed. The accuracy of the Nomogram was estimated in the training and validation groups. A *p*-value of <0.05 was considered statistically significant. Statistical analyses were performed with IBM SPSS (version 23.0), MedCalc (version 19.3), and R statistical software (version 4.0.4).

## Results

### Patient Characteristics and bpMRI Results

**Figure 1:**
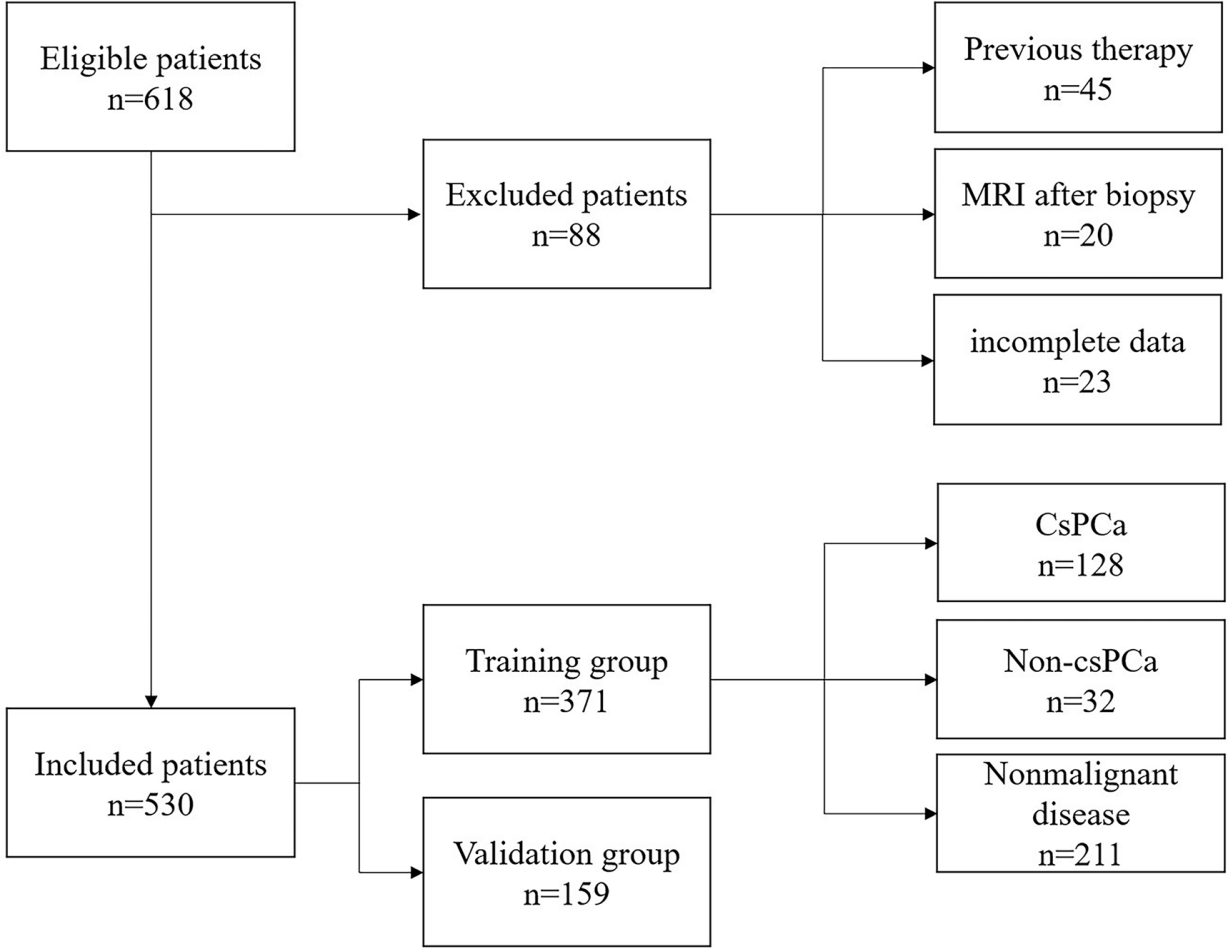
Flow diagram of the inclusion and exclusion criteria in this research.

A total of 530 patients who met the inclusion criteria were enrolled in this study. **Figure 1** is a flowchart that describes how patients and data were selected from the hospital information system (HIS). The training group and validation group consisted of 371 (70%) and 159 (30%) men, respectively, and there was no statistical difference in data characteristics between the two groups (*p* < 0.05). In the training group, 160 (43.1%) patients had PCa, namely, 128 (34.5%) with csPCa and 32 (8.6%) with low-grade PCa (Gleason score = 6) based on the results from both targeted and systemic biopsies (**Table 1**). Regarding the clinical indicators, age (69.85 ± 8.07 *vs.* 65.86 ± 8.33 years, *p* < 0.001), tPSA [16.63 (8.10–24.66) *vs.* 10.00 (7.09–15.57) ng/ml, *p* < 0.001], PV [34.41 (24.70–48.01) *vs.* 47.42 (32.05–67.10) ml, *p* < 0.001], f/tPSA [0.10 (0.07–0.13) *vs.* 0.15 (0.11–0.18), *p* < 0.001], and PSAD [0.46 (0.26–0.72) *vs.* 0.21 (0.14–0.33) ng/ml, *p* < 0.001] were significantly higher in csPCa patients compared with non-csPCa (low-grade PCa and non-malignant disease) (**Table 1**).

**Table 1:** Patient demographics and clinical characteristics of the training group and the validation group.

Indicators	Training Group (*n* = 371)				Validation Group (*n* = 159)			
	CsPCa(*n* = 128)	Non-csPCa (*n* = 32)	Benign lesion (*n* = 211)	*p-*value	CsPCa (*n* = 47)	Non-csPCa (*n* = 16)	Benign lesion (*n* = 96)	*p-*value
Age	69.85 ± 8.07	66.56 ± 6.39	65.76 ± 8.59	<0.001	70.32 ± 9.24	72.06 ± 1.86	65.64 ± 7.45	0.002
fPSA	1.39 (0.86–2.65)	1.11 (0.73–1.76)	1.36 (0.92–1.99)	0.017	1.36 (0.92–3.28)	1.03 (0.75–1.76)	1.23 (0.82–1.84)	0.174
tPSA	16.63 (8.10–24.66)	10.01 (5.91–15.26)	10.00 (7.19–15–57)	<0.001	9.39 (6.52–41.12)	8.75 (6.19–12.64)	8.76 (6.66–11.72)	0.081
f/tPSA	0.10 (0.07–.013)	0.12 (0.09–0.18)	0.15 (0.11–0.19)	<0.001	0.12 (0.09–0.15)	0.13 (0.09–0.150	0.14 (0.10–0.17)	0.151
PV	34.41 (24.70–48.01)	35.72 (27.45–48.34)	49.46 (33.20–67.50)	<0.001	31.34 (24.26–50.52)	38.29 (29.69–58.28)	42.29 (30.83–54.36)	0.024
PSAD	0.46 (0.26–0.72)	0.25 (0.17–0.35)	0.21 (0.14–0.32)	<0.001	0.35 (0.25–0.87)	0.24 (0.13–0.34)	0.22 (0.15–0.30)	<0.001
PI-RADS score								
1	0 (0%)	0 (0%)	19 (9.0%)	<0.001	0 (0.0%)	0 (0%)	6 (6.2%)	<0.001
2	2 (1.6%)	4 (12.5%)	87 (41.3%)		1 (2.1%)	1 (6.3%)	36 (37.5%)	
3	18 (14.1%)	12 (37.5%)	71 (33.6%)		6 (12.8%)	8 (50%)	36 (37.5%)	
4	65 (50.7%)	(34.4%)	28 (13.3%)		30 (63.8%)	5 (33.3%)	17 (17.7%)	
5	43 (33.6%)	5 (15.6)	6 (2.8%)		10 (21.3%)	2 (12.5%)	1 (1.1%)	

fPSA, free PSA; tPSA, total PSA; f/tPSA, free/total PSA; PV, prostate volume; PSAD, PSA density; csPCa, clinical significance prostate cancer; PI-RADS score, prostate imaging reporting and date system score.

According to the bpMRI results, patients with PI-RADS score not less than three in the training group had a 59.5% (154/259) risk of PCa, compared to men with a lower score 5.4% (6/112). In addition, a total of 112 cases were scored as 1 and 2, namely, 2 cases of csPCa, 4 cases of low-grade PCa, and 106 cases of non-malignant lesions. A total of 101 cases were scored as 3, namely, 18 cases of csPCa, 12 cases of low-grade PCa, and 71 cases of non-malignant lesions. Regarding PI-RADS score 4 and 5, there were 108 cases of csPCa, 16 cases of non-csPCa, and 34 cases of non-malignant lesions (**Table 1**). If the cutoff value for biopsy decision is set as PI-RADS < 4, 83.1% (177/213) of patients could avoid biopsy, at the price of missing 11.3% (20/177) csPCa cases (**Table 1**). According to our results, it was apparent that a Total score of 6 was the optimal threshold for detecting csPCa according to ROC curve analysis and Youden’s index calculation; 87.2% (163/187) of patients with a Total score of 2–5 could avoid biopsy at the price of missing 6.9% (13/187) csPCa cases (**Table 4A**).

### ROC Curve Analysis and Prediction Models Establishment

According to ROC curve analysis, the AUCs for diagnosing PCa and csPCa were summarized in **Table 2**. Total score achieved the highest diagnostic accuracy [AUC = 0.903 (0.872–0.933), *p* < 0.001] in distinguishing patients with or without csPCa compared with other indicators, and had 80.5% sensitivity and 86.4% specificity. Regarding PSA derivatives, PSAD has the best diagnostic accuracy in predicting PCa [AUC = 0.734 (0.682–0.787), *p* < 0.001] and csPCa [AUC = 0.765 (0.711–0.819), *p* < 0.001] compared with other PSA derivatives (**Table 2**). In univariable analysis, all clinical indicators were significant predictors for PCa and csPCa, except for fPSA (*p* > 0.05) (**Table 1**). Spearman analysis showed that age, fPSA, tPSA, PSAD, PI-RADS score, and Total score were significantly correlated with Gleason score; Total score was most strongly associated with the malignancy of PCa (**Table 2**).

**Table 2:** Relationship between prebiopsy clinical indicators and Gleason score and evaluating the diagnostic performance of each variable for predicting PCa and csPCa using receiver operating characteristic curve analysis.

Indicators	Relationship between clinical indicators and Gleason Score		ROC curve analysis for diagnosis of PCa			ROC curve analysis for diagnosis of csPCa		
	*R*	*p-*value	AUC	95% CI	*p-*value	AUC	95% CI	*p*-value
Age	0.245	<0.001	0.616	0.559–0.673	<0.001	0.645	0.586–0.704	<0.001
f-PSA	0.067	0.199	0.502	0.441–0.563	0.952	0.534	0.469–0.600	0.276
t-PSA	0.282	< 0.001	0.628	0.569–0.688	<0.001	0.668	0.606–0.730	<0.001
f/t-PSA	−0.323	< 0.001	0.310	0.257–0.365	<0.001	0.296	0.242–0.351	<0.001
PV	−0.275	<0.001	0.325	0.270–0.380	<0.001	0.337	0.280–0.394	<0.001
PSAD	0.442	< 0.001	0.734	0.682–0.787	<0.001	0.765	0.711–0.819	<0.001
PI-RADS score	0.654	< 0.001	0.863	0.825–0.900	<0.001	0.864	0.827–0.901	<0.001
T2WI score	0.632	<0.001	0.848	0.809–0.887	<0.001	0.851	0.812–0.889	<0.001
DWI score	0.667	< 0.001	0.856	0.818–0.894	<0.001	0.859	0.821–0.897	<0.001
Total score	0.664	< 0.001	0.900	0.869–0.931	<0.001	0.903	0.872–0.933	<0.001

fPSA, free PSA; tPSA, total PSA; f/tPSA, free/total PSA; PV, prostate volume; PSAD, PSA density; PI-RADS score, prostate imaging reporting and date system score; T2WI score, T2WI score referring to PI-RADS v2; DWI score, DWI score referring to PI-RADS score; csPCa, clinical significance prostate cancer; Total score, T2WI score + DWI score.

**Figure 2:**
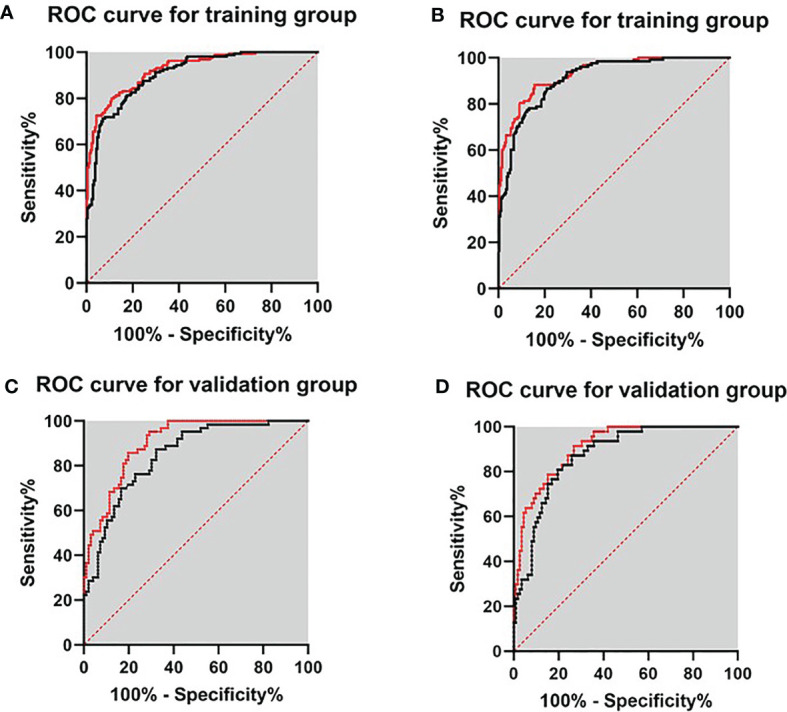
ROC curves for validating the discrimination of two prediction models in the training group and validation group. **(A)** Diagnosis of PCa in the training group. **(B)** Diagnosis of csPCa in the training group. **(C)** Diagnosis of PCa in the validation group. **(D)** Diagnosis of csPCa in the validation group.

To further construct a predictive model for biopsy decision, we performed a stepwise multivariable logistic regression analysis to eliminating the redundant variables and avoiding multicollinearity. Finally, two prediction models were established. F/tPSA, PSAD, and PI-RADS score were incorporated in model 1 (each *p* < 0.001); f/tPSA, PSAD, and Total score were incorporated in model 2 (each *p* < 0.001) for detection of PCa and csPCa, respectively (**Table 3**). Regarding csPCa detection rate with prediction models, the AUCs of model 1 and model 2 were 0.910 (0.881–0.939, *p* < 0.001) and 0.931 (0.906–0.956, *p* < 0.001) for the training group and 0.878 (0.817–0.926, *p* < 0.001) and 0.910 (0.865–0.954, *p* < 0.001) for the validation group (**Figure 2**). The ROC curve analysis showed that model 2 was significantly better than model 1 in both the training group and the validation group according to Delong test (**Supplementary Table S2**). Model 2 had 88.3% sensitivity and 84.4% specificity for diagnosing csPCa. Using the model 2, 85.5% (188/220) of the study patients would have avoided biopsies and 15 csPCa (6.8%,15/220) would have been missed. Sensitivity, specificity, PPV, NPV, and AUC of PSAD; Total score; and two prediction models in the detection of csPCa are reported in **Table 4**.

Table 3aMultivariate stepwise logistic regression analysis for predicting csPCa in the training group.

**Table:** 

Indicators	*B*	SE	*p*	VIF	*t*
PSAD	0.257	0.051	<0.000	1.209	0.507
f/tPSA	−1.160	0.305	<0.000	1.126	−3.806
PIRADS score	0.220	0.017	<0.000	1.103	12.876
Constants	−0.303	0.075	<0.000	-	−4.033

**Table 3b:** Multivariate stepwise logistic regression analysis for predicting csPCa in the training group.

Indicators	*B*	SE	*p*	VIF	*t*
PSAD	0.211	0.049	<0.000	1.236	4.338
f/tPSA	−1.031	0.289	<0.000	1.130	−3.562
Total score	0.140	0.009	<0.000	1.141	15.044
Constants	−0.387	0.072	<0.000	-	−5.393

Model 1: Logit(csPa) = −0.303 + 0.257*PSAD − 1.160*f/tPSA + 0.220*PI-RADS score; Model 2: Logit(csPCa) = −0.387 + 0.211*PSAD − 1.031*f/tPSA + 0.140*Total score. PSAD, PSA density; f/tPSA, free/total PSA; PI-RADS score, prostate imaging reporting and date system score; T2WI score, T2WI score referring to PI-RADS v2; DWI score, DWI score referring to PI-RADS score; Total score, T2WI scored score; B, regression coefficient; SE, standard error; VIF, variance inflation factor.

Table 4aComparison of the diagnostic performance of prediction models and clinical indicators in the training group (csPCa).

**Table:** 

Indicators	AUC	95% CI	Cutoff	Sensitivity	Specificity	PPV	NPV	*p*
t-PSA	0.668	0.606–0.730	14.115	0.641	0.691	52.23%	78.50%	<0.000
PSAD	0.765	0.711–0.819	0.400	0.563	0.856	67.29%	78.19%	<0.000
PIRADS score	0.864	0.827–0.901	3	0.844	0.794	48.65%	92.81%	<0.000
T2WI score	0.848	0.809–0.887	3	0.789	0.790	46.87%	93.41%	<0.000
DWI score	0.859	0.821–0.897	2	0.828	0.778	42.67%	100.00%	<0.000
Total score	0.903	0.872–0.933	6	0.805	0.864	62.60%	93.05%	<0.000
Model 1	0.910	0.881–0.939	0.381	0.867	0.790	68.10%	91.83%	<0.000
Model 2	0.931	0.906–0.956	0.391	0.883	0.844	74.83%	93.18%	<0.000

tPSA, total PSA; PSAD, PSA density; PI-RADS score, prostate imaging reporting and date system score; T2WI score, T2WI score referring to PI-RADS v2; DWI score, DWI score referring to PI-RADS score; csPCa, clinical significant prostate cancer; Total score, T2WI score + DWI score. Model 1 = f/tPSA + PSAD + T2WI score. Model 2 = f/tPSA + PSAD + Total score; AUC, area under the curve; 95% CI, 95% confidence interval for AUC; Cutoff, best cutoff; PPV, positive predictive value; NPV, negative predictive value.

**Table 4b:** Comparison of the diagnostic performance of prediction models and clinical indicators in the validation group (csPCa).

Indicators	AUC	95% CI	Cutoff	Sensitivity	Specificity	PPV	NPV	*p*
t-PSA	0.610	0.501–0.718	13.480	0.383	0.92	64.29%	77.86%	0.047
PSAD	0.733	0.643–0.823	0.290	0.660	0.732	50.00%	83.51%	<0.000
PIRADS score	0.847	0.785–0.910	3	0.851	0.777	40.00%	97.73%	<0.000
T2WI score	0.815	0.747.0.883	3	0.767	0.901	38.87%	94.76	<0.000
DWI score	0.830	0.763–0.897	3	0.766	0.777	36.72%	100.00%	<0.000
Total score	0.888	0.838–0.938	5	0.914	0.714	43.93%	100.00%	<0.000
Model 1	0.872	0.817–0.926	0.944	0.872	0.741	57.75%	93.18%	<0.000
Model 2	0.910	0.865–0.954	1.101	0.915	0.732	58.90%	95.35%	<0.000

tPSA, total PSA; PSAD, PSA density; PI-RADS score, prostate imaging reporting and date system score; T2WI score, T2WI score referring to PI-RADS v2; DWI score, DWI score referring to PI-RADS score; csPCa, clinical significant prostate cancer; Total score, T2WI score + DWI score. Model 1 = f/tPSA + PSAD + T2WI score. Model 2 = f/tPSA + PSAD + Total score; AUC, area under the curve; 95% CI, 95% confidence interval for AUC; Cutoff, best cutoff; PPV, positive predictive value; NPV, negative predictive value.

### DCA and Comparison of the Net Benefit of Models and Clinical Indicators

**Figure 3:**
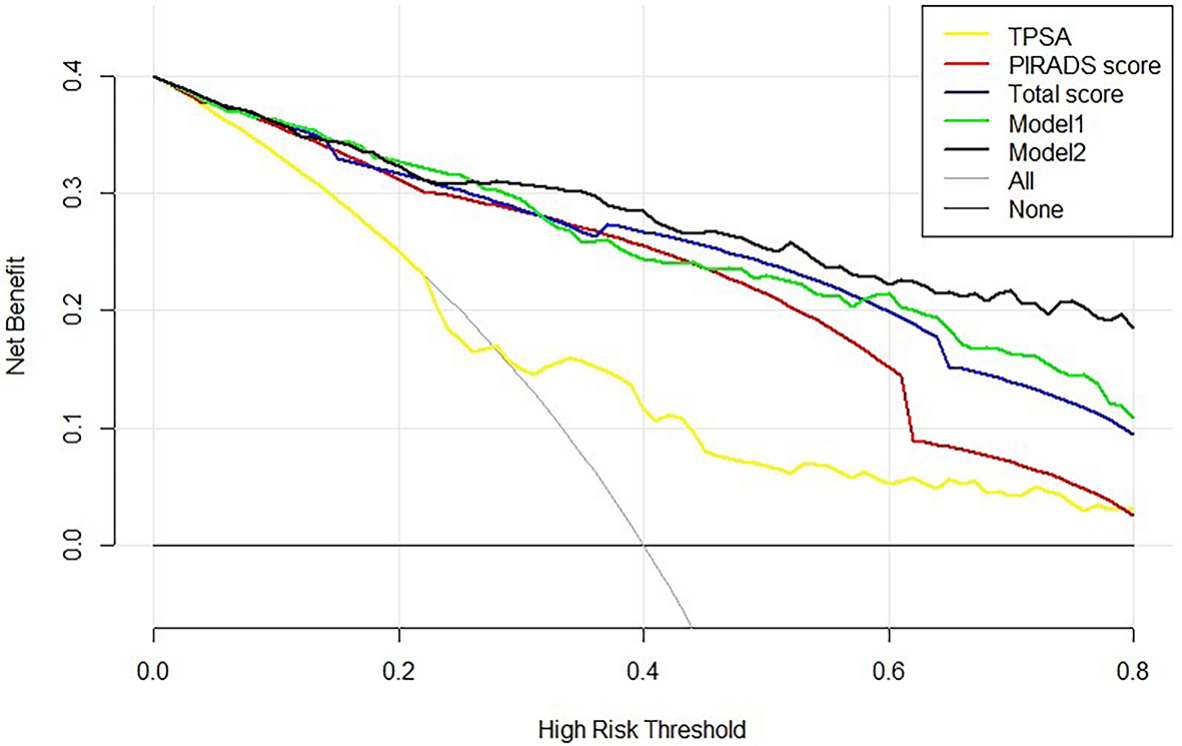
Decision curve analysis of the PSA, bpMRI score, and prediction models for csPCa. The net benefit curves for models are shown in this figure. The *x*-axis indicates the threshold probability for critical care outcome and the *y*-axis indicates the net benefit. For the baselines, solid transverse line = net benefit when all patients are considered as not having the outcome; dashed line = net benefit when all patients are considered as having the outcome. Solid black line = model 2, solid green line = model 1, solid blue line = Total score, solid red line = PI-RADS score, and solid yellow line = PSA. The preferred model is model 2, the net benefit of which was highest over the range of other parameters. Model 2 with the greatest net benefit at a given risk threshold had the greatest clinical value.

To assess the potential clinical benefit of the two models and clinical indicators, we performed DCA using the predicted risk in the training group and validation group. The overall utility of the decision models (i.e., PSA, PI-RADS score, Total score, model 1, and model 2) were examined (**Figure 3** and **Supplemental Figure S1**). Two models showed a higher net benefit than PSA when the threshold probability was 0.05–0.80. Model 2 showed the highest net benefit with threshold probabilities > 0.25, superior to other indicators. Assuming we choose to diagnose and treat csPCa with a predicted probability of 20%, for every 100 men using Model 2, 32 will benefit without detriment to anyone else. For every 100 men who use PSA, only 25 men benefit and without detriment to anyone else. If the threshold probability > 20%, Model 2 also has a better net benefit than other indicators such as PI-RADS score and Total score (**Figure 3**). For example, applying a biopsy risk threshold of 40% meant that 57.1% of all men could avoid a biopsy; clinical impact curve (CIC) visually indicated the high clinical net benefit and confirmed the clinical value of model 2 (**Supplemental Figure S2**).

### Internal Validation of the Nomogram for Predicting csPCa

**Figure 4:**
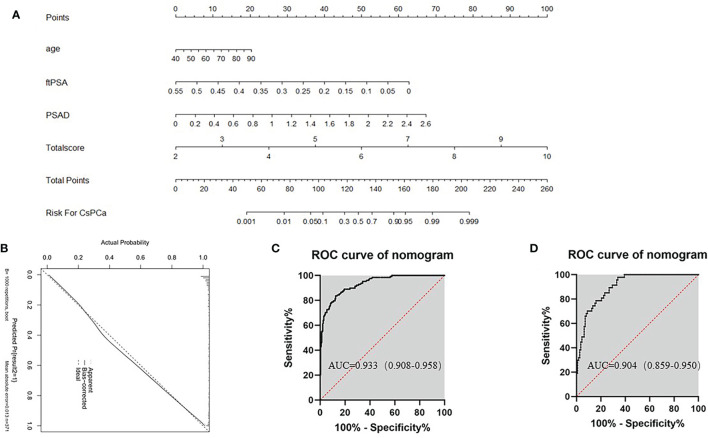
Nomogram predicts the probability of csPCa. Established a Nomogram based on bpMRI and other clinical indicators. **(A)** Nomogram for diagnosis of clinically significant cancer (csPCa). Higher total points indicated a higher prevalence for csPCa. **(B)** Nomogram-predicted probability of csPCa. **(C)** The AUC of Nomogram-predicted probability of csPCa in the training group. **(D)** The AUC of Nomogram-predicted probability of csPCa in the validation group.

In order to verify the predictive ability of Total score and Model 2, the Nomogram constructed by age, f/tPSA, PSAD, and Total score was established (**Figure 4**). Points from each variable are added and a straight line from the total point score shows the probability of harboring csPCa. Combining the Total score with clinical factors increased the AUC for diagnosis of csPCa (AUC = 0.933). Furthermore, the calibration curves were depicted in the training group to estimate the agreement between the estimated risk of Nomogram and the actual csPCa risk. In the validation group, we verified the diagnostic performance of Nomogram in csPCa and the AUC reached 0.904. We using bootstrap method and calibration plots also confirmed the stability of the Nomogram.

## Discussion

Currently, prostate biopsy is still the gold standard in diagnosing PCa. Though PSA is a widely accepted biomarker for PCa, it is not ideal and often criticized for leading to unnecessary biopsy. The use of PSA derivatives such as fPSA, f/tPSA, PSAD, and PSA velocity (PSAV) may improve the diagnostic accuracy of PCa (26), but the improvement in clinical benefits was still limited. In addition, several novel biomarkers including Prostate Health Index (PHI), Prostate Cancer Antigen 3 (PCA3), *4K Score*, and a number of non-coding RNAs have been reported in improving the diagnosis accuracy of PCa, especially at initial biopsy (27–30), but most of them are not widely used at present.

The important role of MRI in the diagnosis of PCa was confirmed in the PRECISION study (31). MRI-based parameters are reflective of pathologically determined characteristics of PCa. Abnormal MRI performance is positively associated with increased tumor volume and higher tumor grade (32), and this is consistent with our research results (**Table 2**). The purpose of the DCE sequence of mpMRI is to assess tumor angiogenesis; however, uncertainty still exists about the added value or cost-effectiveness (33). In the version of PI-RADS 2.1, the role of T2WI and DWI sequences was more emphasized, and DCE plays a minor role in the assessment of the transitional zone of the prostate gland (34). A previous IMPROD trial demonstrated that bpMRI achieved a high sensitivity for the detection of malignant lesions, especially for csPCa (35). Simultaneously, bpMRI is a less-invasive imaging modality and functions as a rapid, affordable screening tool for PCa screening. Rosenkrantz et al. found that T2WI combined with DWI had higher sensitivity than T2WI alone, but the addition of DCE did not improve the diagnostic sensitivity (36). A meta-analysis reported that bpMRI was only 7% less sensitive than mpMRI in the diagnosis of PCa (37). In another meta-analysis, Woo et al. found that the performance of bpMRI was similar to that of mpMRI in the diagnosis of PCa (38). The diagnostic accuracy of bpMRI is comparable with that of mpMRI in the detection of PCa and the identification of csPCa. Thus, bpMRI may serve as a faster, cheaper, and contrast agent-free alternative to mpMRI.

According to previous studies, a PI-RADS score of 3 was found in 31%–32% of post-biopsy patients (39, 40). Of particular note, although the PI-RADS protocol provides detailed grading criteria, the guidelines do not describe how to deal with a PI-RADS score of 3 (13). Robertson et al. modified the PI-RADS score of MRI to increase the PI-RADS score by 3 to 4 scores based on the significant performance of a particular sequence in mpMRI (41). In the review of our study, the detection rate of PCa and csPCa in patients with a PI-RADS score of 3 was 29.1% (44/151) and 15.9% (24/151) respectively, which is consistent with previous research (40, 42, 43). Considering that the diagnosis is ambiguous for patients with a PI-RADS score of 3, we evaluated bpMRI performance by summation of T2WI score and DWI score and found that when PI-RADS score = 3 and Total score > 5, the detection rate of csPCa was 62.5% (**Table 4**), which provides important information for biopsy decision-making.

In this study, we further quantified the bpMRI parameters and provided good performance for identifying csPCa (AUC = 0.903). We evaluated prostate lesions on bpMRI and found that T2WI score and Total score alone could diagnose 68.4% and 75.7% of csPCa, respectively (**Table 4**). Despite the high sensitivity of T2WI score in the diagnosis of csPCa (**Table 4**), benign lesions like BPH, prostatic nodules, and prostatitis can also present as low signal appearance (44, 45). Regarding the bpMRI results, the Total score for PCa and csPCa detection achieved the highest prediction accuracy in both the training group and the validation group, and the AUCs were significantly higher than the PI-RADS score of bpMRI reported in other studies (0.771–0.830) (21, 46, 47).

Although the diagnostic specificity of PSA is poor, recent studies have shown that PSAD is an independent predictor of PCa compared with PSA or PSA derivatives (4, 48). Falagario et al. (49) used 10 strategies with combinations of bpMRI scoring and PSAD, but still had a 10% risk of missing csPCa. Using model 2 of our study, 85.5% of the patients would have avoided biopsies, and only 6.8% of csPCa were missed. Our study also confirms the additional role of PSAD in the diagnosis of PCa (**Table 4**). A prior systematic study also showed that f/tPSA < 0.15 had a relative high sensitivity and specificity with tPSA levels in 2–10 ng/ml (50). Then, f/tPSA was considered to be an important predictor in multivariate analysis, which led to analysis of combined bpMRI, f/tPSA, and PSAD in this study. Our study showed that the combination of the Total score, PSAD, and f/tPSA could improve the diagnosis of csPCa (AUC = 0.931), and had a sensitivity and specificity of 88.3% and 84.4%, respectively, which is significantly better than the predictive power of either the PI-RADS score (AUC =0.863) and other indicators. ROC curve analysis shows that the AUC was significantly enhanced when Total score was combined with f/tPSA and PSAD in this study.

There are also limitations of this study. First, the main limitation was the use of biopsy results as a reference standard, clinically significant lesions could have been missed, and the true rate of false negatives cannot be evaluated because post-prostatectomy specimens of patients were unknown. Second, the measurement of PV according to T2WI sequence results lacks contrast agent enhancement examination of DCE, which may reduce the bpMRI scoring and affect the detection rate of csPCa. Third, due to the limited sample size, patients with PSA gray zone (4–10 ng/ml) were not individually analyzed; further studies that include more patient information are ongoing. Finally, implicit bias may exist due to the single-center retrospective nature of the study, and multicenter studies will be carried out to confirm our findings.

In conclusion, this study not only validates the results of previous studies about bpMRI but also demonstrates that using a convenient, rapid, and less expensive bpMRI-based prediction model can achieve better diagnostic accuracy. Both the prediction model and Nomogram established in this study can be further used to guide clinical decision-making. It is hoped that in the future, a risk calculator, which contains T2WI, DWI, patient’s age, f/tPSA, prostate volume, and constructs based on our Nomogram, will be used as a computer program or smartphone application to guide biopsy more conveniently.

## Data Availability Statement

The original contributions presented in the study are included in the article/**Supplementary Material**. Further inquiries can be directed to the corresponding authors.

## Ethics Statement

The studies involving human participants were reviewed and approved by Ethics Review Committee of Ningbo First Hospital. Written informed consent for participation was not required for this study in accordance with the national legislation and the institutional requirements.

## Author Contributions

J-FP and QM conceptualized, designed, and supervised the study. J-FP, J-ZC, R-DH, Z-LT, and C-LY coordinated and participated in data collection. J-FP, RS, S-ZY, and QM carried out the statistical analysis and drafted the manuscript. RS performed a prostate biopsy. Z-YZ and D-WR participated in the image scoring of bpMRI. QM provided guidance and revised the manuscript. All authors contributed to the article and approved the submitted version.

## Funding

This study was supported by the Zhejiang Natural Science Fund (Grant No. LY20H050002 to QM, Grant No. LY18H050003 to J-HJ), the Medical Health Science and Technology Project of Zhejiang Provincial Health Commission (Grant No. 2021KY977 to RS), the Ningbo Social Development Fund (Grant No. 202002N3192 to QM), and the Fund of Ningbo Clinical Research Center for Urological Disease (2019A21001).

## Conflict of Interest

The authors declare that the research was conducted in the absence of any commercial or financial relationships that could be construed as a potential conflict of interest.

## Publisher’s Note

All claims expressed in this article are solely those of the authors and do not necessarily represent those of their affiliated organizations, or those of the publisher, the editors and the reviewers. Any product that may be evaluated in this article, or claim that may be made by its manufacturer, is not guaranteed or endorsed by the publisher.

## Supplementary Material

Supplementary Figure 1 Decision curve analysis of the PSA, bpMRI parameters and two prognostic models for PCa and csPCa in the training group and validation group. The net benefit curves for models and parameters are shown in this figure. X-axis indicates the threshold probability for critical care outcome and Y-axis indicates the net benefit. Solid black line = model 2, solid green line = model 1, solid red line = PI-RADS score, solid blue line = Total score and solid yellow line = PSA. The preferred model is the model 2, the net benefit of which was larger over the range of other parameters. For the base lines, solid transverse line = net benefit when all patients are considered as not having the outcome; dashed line = net benefit when all patients are considered as having the outcome. The model with the greatest net benefit at a given risk threshold had the greatest clinical value. **(A)**: DCA for training group (PCa) **(B)**: DCA for validation group (csPCa) **(C)**: DCA for validation group (PCa).

Supplementary Figure 2 Clinical impact curve (CIC) of model 2 in training group and validation group. **(A)**: Clinical impact curve for training group; **(B)**: Clinical impact curve for validation group. The red curve (number of high-risk individuals) indicates the number of people who are classified as positive (high risk) by the model at each threshold probability; the blue curve (number of high-risk individuals with outcome) is the number of true positives at each threshold probability. CIC visually indicated that nomogram conferred high clinical net benefit and confirmed the clinical value of the model 2.

Supplementary Table 1 BpMRI PI-RADS v.2 score criteria and Total score scoring criteria **(A)** PI-RADS Assessment Category for the Peripheral Zone (PZ). **(B)** PI-RADS Assessment Category for the Transition Zone (TZ). **(C)** PI-RADS Assessment for Peripheral Zone on T2-Weighted Imaging. **(D**) PI-RADS Assessment for Transition Zone on T2-Weighted Imaging. **(E)** PI-RADS Assessment for DWI for both Peripheral Zone and Transition Zone

Supplementary Table 2 DeLong test compared Model 1 and Model 2 to diagnose csPCa in the training group and the validation group, respectively. *AUC, area under the curve; SE, standard error.*

Supplementary Table 3 The pathology findings of the patients with csPCa in the two groups. Biopsy specimens were reviewed by experienced pathologists according to the ISUP 2014 modified Gleason score (GS)/Grade Group (G) system: GG1, Gleason score 3 + 3; GG2, Gleason score 3 + 4 and; GG3, Gleason score 4 + 3; GG4, Gleason score include 3 + 5, 4 + 4 and 5 + 3; GG5, Gleason score include 4 + 5, 5 + 4 and 5 + 5. And Gleason score of 3 + 4 or higher was defined as csPCa.
