# Supplementary material for: Modified Predictive Model and Nomogram by Incorporating Prebiopsy Biparametric Magnetic Resonance Imaging With Clinical Indicators for Prostate Biopsy Decision Making
Source: Front Oncol. 2021 Sep 13;11:740868. doi: 10.3389/fonc.2021.740868 (PMC8473816; doi:10.3389/fonc.2021.740868)
Supplement: Supplementary Figure 1 — Decision curve analysis of the PSA, bpMRI parameters and two prognostic models for PCa and csPCa in the training group and validation group. The net benefit curves for models and parameters are shown in this figure. X-axis indicates the threshold probability for critical care outcome and Y-axis indicates the net benefit. Solid black line = model 2, solid green line = model 1, solid red line = PI-RADS score, solid blue line = Total score and solid yellow line = PSA. The preferred model is the model 2, the net benefit of which was larger over the range of other parameters. For the base lines, solid transverse line = net benefit when all patients are considered as not having the outcome; dashed line = net benefit when all patients are considered as having the outcome. The model with the greatest net benefit at a given risk threshold had the greatest clinical value. (A): DCA for training group (PCa) (B): DCA for validation group (csPCa) (C): DCA for validation group (PCa). [file DataSheet_1.zip › Supplementary_Material.docx]

Supplementary Material

# Supplementary Figures and Tables

## Supplementary Figures


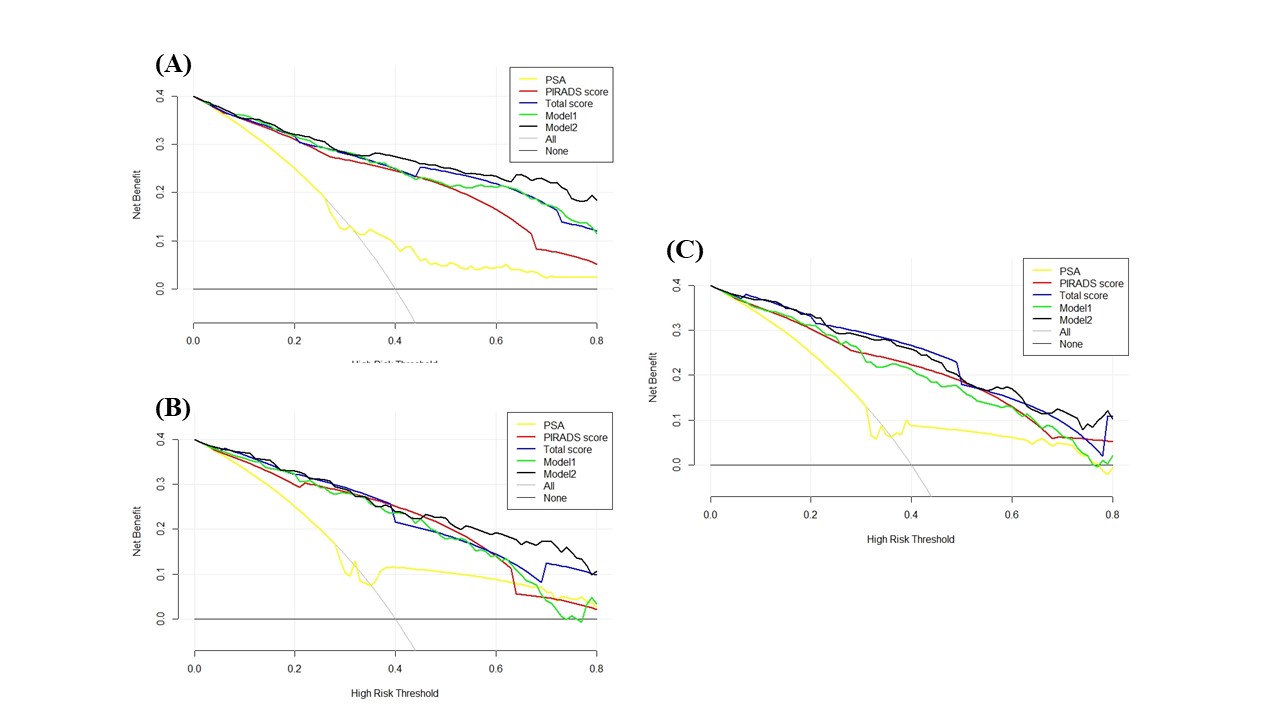


**Supplementary Figure 1.** The net benefit curves for models and parameters are shown in this figure. X-axis indicates the threshold probability for critical care outcome and Y-axis indicates the net benefit. Solid black line = model 2, solid green line = model 1, solid red line = PIRADS score, solid blue line = Total score and solid yellow line = PSA. The preferred model is the model 2, the net benefit of which was larger over the range of other parameters. For the base lines, solid transverse line = net benefit when all patients are considered as not having the outcome; dashed line = net benefit when all patients are considered as having the outcome. The model with the greatest net benefit at a given risk threshold had the greatest clinical value. (A): DCA for training group (PCa) (B): DCA for validation group (csPCa) (C): DCA for validation group (PCa).


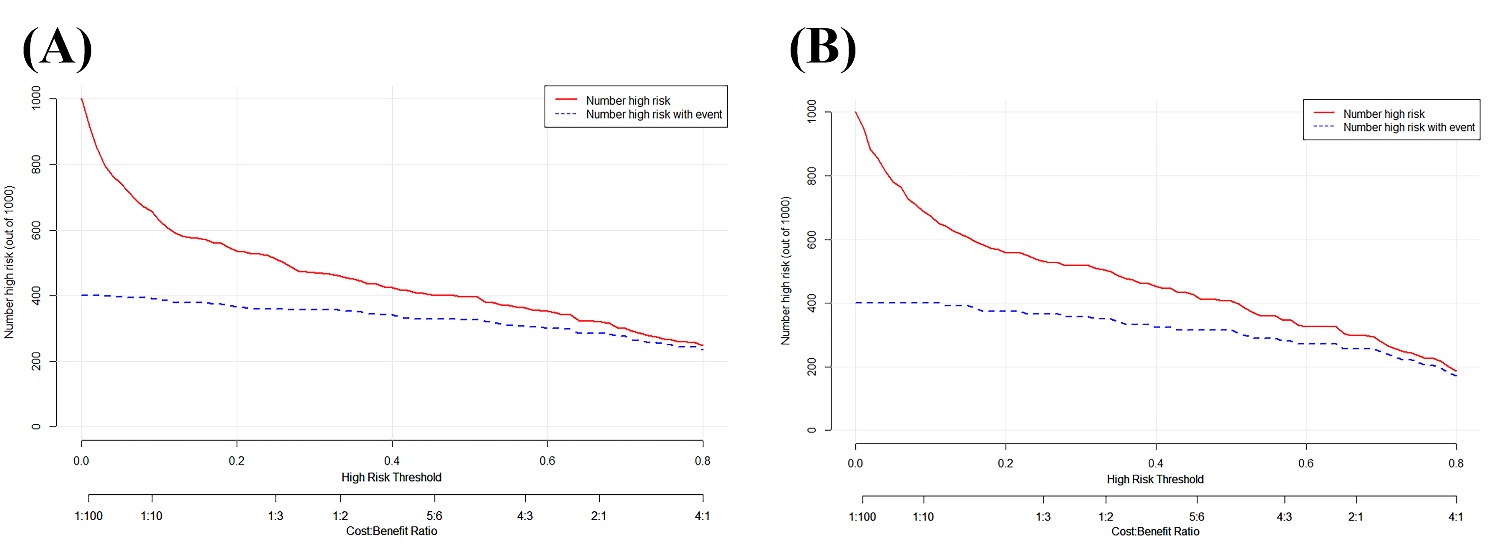


**Supplementary Figures 2.**  Clinical impact curve (CIC) of model 2 in training group and validation group. (A): Clinical impact curve for training group; (B): Clinical impact curve for validation group. The red curve (number of high-risk individuals) indicates the number of people who are classified as positive (high risk) by the model at each threshold probability; the blue curve (number of high-risk individuals with outcome) is the number of true positives at each threshold probability. CIC visually indicated that nomogram conferred high clinical net benefit and confirmed the clinical value of the model 2.

## Supplementary Tables

**Supplemental Table 1.** BpMRI PI-RADS v.2 score criteria and Total score scoring criteria

**(a) PI-RADS Assessment Category for the Peripheral Zone (PZ)**

| T2WI | DWI | DCE | PI-RADS v2 | Total score |
| --- | --- | --- | --- | --- |
| Any | 1 | / | 1 | 1 + Any |
| Any | 2 | / | 2 | 2 + Any |
| Any | 3 | / | 3 | 3 + Any |
| Any | 4 | / | 4 | 4 + Any |
| Any | 5 | / | 5 | 5 + Any |

**(b) PI-RADS Assessment Category for the Transition Zone (TZ)**

| T2WI | DWI | DCE | PI-RADS v2 | Total score |
| --- | --- | --- | --- | --- |
| 1 | Any | / | 1 | 1 + Any |
| 2 | Any | / | 2 | 2 + Any |
| 3 | <5 | / | 3 | 3 + Any |
| 3 | 5 | / | 4 | 3 + 5 |
| 4 | Any | / | 4 | 4 + Any |
| 5 | Any | / | 5 | 5 + Any |

**(c) PI-RADS Assessment for Peripheral Zone on T2-Weighted Imaging**

| **Score** | **imaging finding** |
| --- | --- |
| 1 | Uniform hyperintense signal intensity(normal). |
| 2 | Linear, wedge-shaped, or diffuse mild hypointensity, usually indistinct margin. |
| 3 | Heterogeneous signal intensity or non-circumscribed, rounded, moderate hypointensity |
| 4 | Circumscribed, homogeneous moderate hypointense focus/mass confined to prostate and <1.5 in greatest dimension |
| 5 | Same as 4, but ≥1.5cm in greatest dimension or definite extraprostatic extension/invasive behavior. |

**(d) PI-RADS Assessment for Transition Zone on T2-Weighted Imaging**

| **Score** | **imaging finding** |
| --- | --- |
| 1 | Homogeneous intermediate signal intensity(normal). |
| 2 | Circumscribed, hypointense or heterogeneous encapsulated nodule(s)(BPH) |
| 3 | Heterogeneous signal intensity with obscured margins, Included others that do not qualify as 2,4 or 5 |
| 4 | Lenticulate or circumscribed, homogeneous, moderately hypointense, and <1.5cm in greatest dimension. |
| 5 | Same as 4, but ≥1.5cm in greatest dimension or definite extraprostatic extension/invasive behavior. |

**(e) PI-RADS Assessment for DWI for both Peripheral Zone and Transition Zone**

| **Score** | **imaging finding** |
| --- | --- |
| 1 | No abnormality (i.e., normal) on ADC and high b-value DWI. |
| 2 | Indistinct hypointense on ADC. |
| 3 | Focal mildly/moderately hypointense on ADC and isointense/  mildly hyperintense on high b-value DWI. |
| 4 | Focal markedly hypontense on ADC and markedly hyperintense  on high b-value DWI; <1.5 cm in greatest dimension. |
| 5 | Same as 4 but ≥1.5 cm in greatest dimension or definite  extraprostatic extension/invasive behavior |

**Supplemental Table 2.** The DeLong test compared Model 1 and Model 2 to diagnose csPCa in the training group and the validation group, respectively

| Group | Prediction Model | AUC | SE | Z value | *P* value |
| --- | --- | --- | --- | --- | --- |
| Training Group | Model 1 | 0.910 | 0.0149 | 2.990 | 0.0028 |
|  | Model 2 | 0.931 | 0.0129 |  |  |
|  | Model 2 | 0.931 | 0.0129 | 4.549 | <0.001 |
|  | Total score | 0.903 | 0.0153 |  |  |
|  | T2WI score | 0.864 | 0.0178 | 4.124 | <0.001 |
|  | Total score | 0.903 | 0.0153 |  |  |
| Validation Group | Model 1 | 0.872 | 0.0279 | 2.443 | 0.0146 |
|  | Model 2 | 0.910 | 0.0225 |  |  |
|  | Model 2 | 0.910 | 0.0225 | 1.655 | 0.098 |
|  | Total score | 0.888 | 0.0247 |  |  |
|  | T2WI score | 0.847 | 0.0296 | 2.376 | 0.0175 |
|  | Total score | 0.888 | 0.0247 |  |  |

*AUC,area under the curve; SE,standard error.*

**Supplemental Table 3.** The pathology findings of the patients with csPCa in the two groups.

| Grading Groups | Training group(n=128) | Validation group(n=47) |
| --- | --- | --- |
| GG 1 | 36 | 16 |
| GG 2 | 45 | 18 |
| GG 3 | 31 | 7 |
| GG 4 | 43 | 15 |
| GG 5 | 9 | 7 |

Biopsy specimens were reviewed by experienced pathologists according to the ISUP 2014 modified Gleason score (GS)/Grade Group (G) system: GG1, Gleason score 3+3; GG2, Gleason score 3+4 and; GG3, Gleason score 4+3; GG4, Gleason score include 3+5, 4+4 and 5+3; GG5, Gleason score include 4+5, 5+4 and 5+5. And Gleason score of 3+4 or higher was defined as csPCa.
