# Supplementary figures and images for: Modified Predictive Model and Nomogram by Incorporating Prebiopsy Biparametric Magnetic Resonance Imaging With Clinical Indicators for Prostate Biopsy Decision Making
Source: Front Oncol. 2021 Sep 13;11:740868. doi: 10.3389/fonc.2021.740868 (PMC8473816; doi:10.3389/fonc.2021.740868)

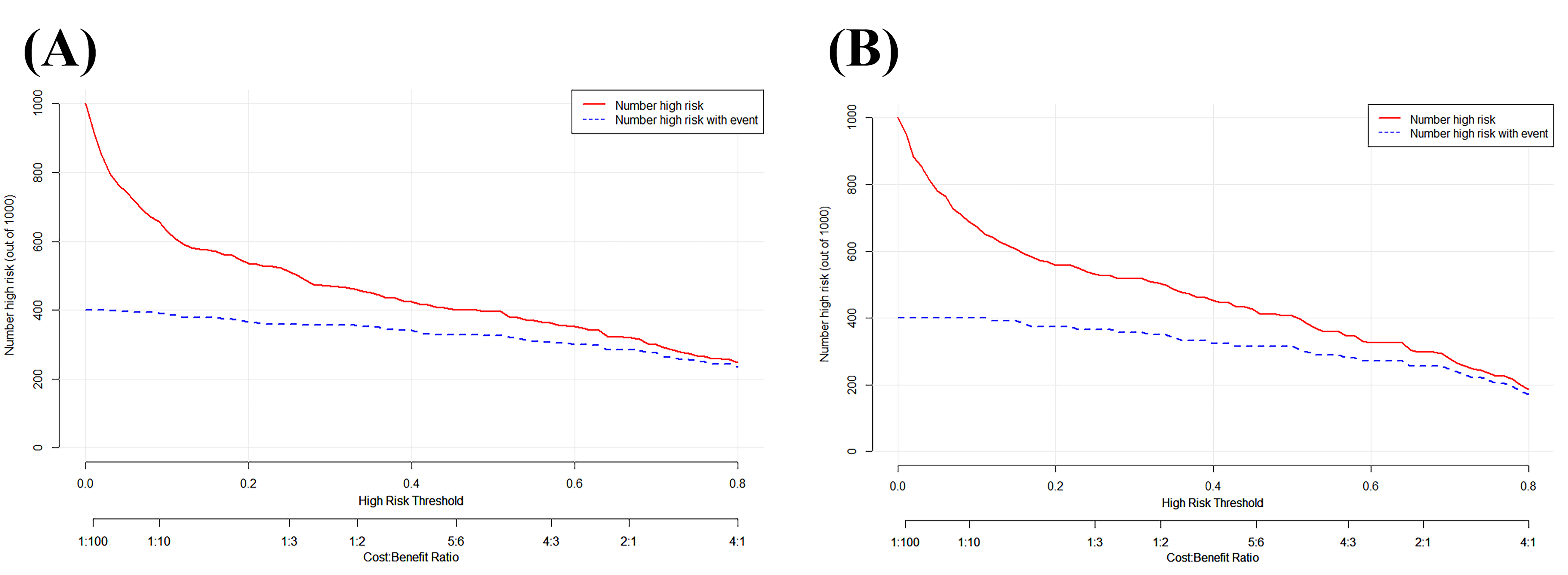

Supplement: Supplementary Figure 1 — Decision curve analysis of the PSA, bpMRI parameters and two prognostic models for PCa and csPCa in the training group and validation group. The net benefit curves for models and parameters are shown in this figure. X-axis indicates the threshold probability for critical care outcome and Y-axis indicates the net benefit. Solid black line = model 2, solid green line = model 1, solid red line = PI-RADS score, solid blue line = Total score and solid yellow line = PSA. The preferred model is the model 2, the net benefit of which was larger over the range of other parameters. For the base lines, solid transverse line = net benefit when all patients are considered as not having the outcome; dashed line = net benefit when all patients are considered as having the outcome. The model with the greatest net benefit at a given risk threshold had the greatest clinical value. (A): DCA for training group (PCa) (B): DCA for validation group (csPCa) (C): DCA for validation group (PCa). [file DataSheet_1.zip › Clinical impact curve (CIC) of model 2 in training group and validation group.jpg]

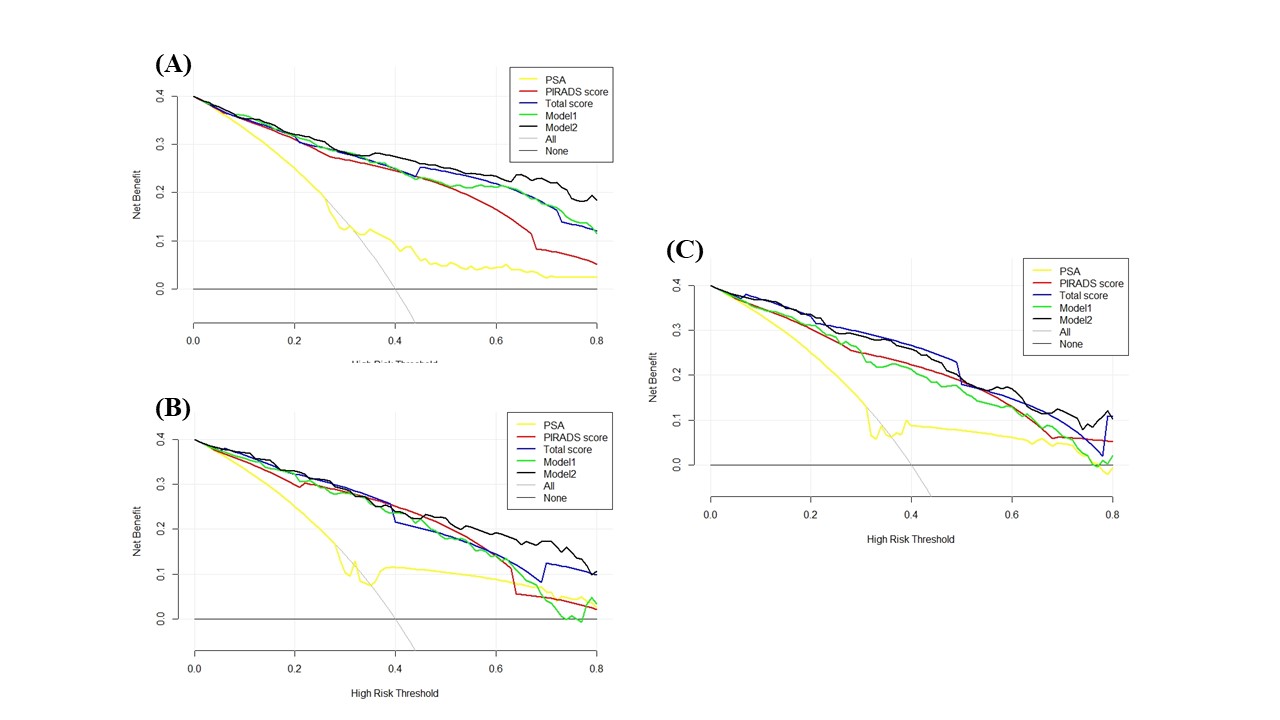

Supplement: Supplementary Figure 1 — Decision curve analysis of the PSA, bpMRI parameters and two prognostic models for PCa and csPCa in the training group and validation group. The net benefit curves for models and parameters are shown in this figure. X-axis indicates the threshold probability for critical care outcome and Y-axis indicates the net benefit. Solid black line = model 2, solid green line = model 1, solid red line = PI-RADS score, solid blue line = Total score and solid yellow line = PSA. The preferred model is the model 2, the net benefit of which was larger over the range of other parameters. For the base lines, solid transverse line = net benefit when all patients are considered as not having the outcome; dashed line = net benefit when all patients are considered as having the outcome. The model with the greatest net benefit at a given risk threshold had the greatest clinical value. (A): DCA for training group (PCa) (B): DCA for validation group (csPCa) (C): DCA for validation group (PCa). [file DataSheet_1.zip › Decision curve analysis of the PSA, bpMRI parameters and two prognostic models for PCa and csPCa in the training group and validation group1.jpg]
